# Effects of fecal microbiota transplantation on clinical outcomes and fecal microbiota of foals with diarrhea

**DOI:** 10.1111/jvim.17185

**Published:** 2024-09-12

**Authors:** Jillian Bell, Sharanne L. Radial, Rosemary S. Cuming, Gareth Trope, Kristopher J. Hughes

**Affiliations:** ^1^ Charles Sturt University School of Agricultural, Environmental and Veterinary Sciences Wagga Wagga New South Wales Australia; ^2^ Scone Equine Hospital Scone New South Wales Australia; ^3^ South Eastern Equine Hospital Narre Warren North Victoria Australia

**Keywords:** foal, next‐generation sequencing, transplant

## Abstract

**Background:**

Diarrhea in foals can be associated with disruption of the intestinal microbiota (dysbiosis). Effective management of intestinal dysbiosis in foals has not been demonstrated.

**Hypothesis/Objectives:**

Fecal microbiota transplantation (FMT) in foals with diarrhea influences the intestinal microbiota and improves clinical and clinicopathological outcomes.

**Animals:**

Twenty‐five foals <6 months of age with diarrhea and systemic inflammatory response syndrome at 3 veterinary hospitals.

**Methods:**

A prospective randomized placebo‐controlled cohort study. Foals in the FMT group (n = 19) or control group (n = 9) received FMT or electrolyte solution once daily for 3 days. Fecal samples were obtained on Day 0 (D0), D1, D2, D3, and D7. Within group and between group data analyses were performed for clinical, clinicopathological, and microbiota variables.

**Results:**

Treatment had no effect on survival (FMT 79%; control 100%, *P* = .3) or resolution of diarrhea (FMT 68%; control 55%, *P* = .4). On D3, the white blood cell count of the FMT group was lower than the control group (D3 FMT group median 6.4 g/L [5‐8.3 g/L]; D3 control group median 14.3 g/L [6.7‐18.9 g/L] *P* = .04). Heart rate reduced over time in the FMT group (D0 median 80 bpm [60‐150 bpm]; D2 median 70 bpm [52‐110 bpm] [*P* = .005]; and D3 median 64, [54‐102 bpm] [*P* < .001]). Phylum Verrucomicrobiota, genus *Akkermansia*, and family *Prevotellaceae* were enriched in the FMT group on D1 (linear discriminate analysis > 4).

**Conclusions and Clinical Importance:**

In foals with diarrhea, FMT appears safe and can be associated with some clinical and microbiota changes suggestive of beneficial effect.

**Abbreviations**
ANOSIM: analysis of similarity
ASV: amplicon sequence variants
D0: day 0
D1: day 1
D2: day 2
D3: day 3
D7: day 7
DNA: deoxyribonucleic acid
ESBL: extended spectrum B lactamase
FMT: fecal microbiota transplantation
LefSe: linear discriminant analysis effect size
PCA: principal component analysis
QIIME2: Quantitative Insights Into Microbial Ecology
WBC: white blood cell

## 1 INTRODUCTION

Diarrhea affects up to 80% of foals in the first 6 months of life^1^, ^2^ and can be associated with noninfectious and infectious causes. Infectious causes of diarrhea in foals include equine rotavirus,^2^ equine coronavirus,^2^ *Clostridioides difficile*,^2^ *Clostridium perfringens*,^2^ *Lawsonia intracellularis*,^3^ *Rhodococcus equi*,^4^ *Salmonella* spp., *Cryptosporidium* spp.,^2^ and *Strongyloides westeri*.^2^, ^4^ Disruption of intestinal microbial communities (dysbiosis) can occur in diarrhea in both adult horses and foals. Differences in the relative abundance of bacteria, reduced alpha‐diversity and increased beta‐diversity are described in adult horses with colitis.^5^, ^6^, ^7^, ^8^, ^9^ Similarly, diarrhea in foals is associated with reduced bacterial richness and diversity, and changes in fecal microbiota composition.^10^, ^11^ Dysbiosis can disrupt intestinal function, energy metabolism, and mucosal health, predispose to inflammation, impair immunity, and reduce resistance to colonization by enteric pathogens. Consequently, restoration of the microbiota is an important objective of the management of horses and foals with diarrhea. Optimal methods for the manipulation of intestinal microbiota are yet to be established.

Treatment of diarrhea in foals is usually nonspecific and supportive, including administration of fluids intravenously and enterally, intraluminal toxin binding agents, and nutritional support.^12^ However, these treatments do not address dysbiosis and there is limited information on microbiota manipulation in foals. Administration of antimicrobial drugs is common in the treatment of diarrhea in foals but is associated with disturbances of the microbiota in adult horses,^13^, ^14^, ^15^ and these changes likely occur in foals as well. Early studies evaluating probiotic administration to foals report no adverse effects^16^, ^17^; however, subsequent studies demonstrate that the administration of probiotic to foals with diarrhea increases the severity of disease,^18^ reduces growth rate^19^, ^20^ and, in healthy foals, is associated with the development of diarrhea requiring veterinary treatment.^18^, ^19^

Fecal microbiota transplantation (FMT) in adult horses reduces diarrhea,^6^, ^7^ increases alpha‐diversity of the fecal microbiota^6^ and results in greater phylogenetic normalization of microbiota.^7^ Conversely, retrospective evaluation of the use of FMT in adult horses reported no improvement in hospitalization duration, fecal consistency, or clinical and clinicopathological variables with treatment.^21^ In addition, FMT administration did not alter the fecal microbiota of horses with diarrhea in another study,^22^ and administration of FMT to horses undergoing treatment with metronidazole failed to prevent or inhibit dysbiosis.^23^ The administration of FMT to adult horses with free fecal water syndrome resulted in no change to the fecal microbiota.^24^ The use of FMT in foals with diarrhea is not reported and it is unknown if this method for manipulation of the intestinal microbiota is safe and effective.

The objective of this study was to investigate the clinical and clinicopathological outcomes and changes in the fecal microbiota associated with the administration of FMT in foals with diarrhea. Our hypothesis was that FMT in foals with diarrhea is associated with improved clinical and clinicopathological outcomes, resolution of diarrhea, and restoration of fecal microbiota.

## 2 MATERIALS AND METHODS

### 2.1 Animals and study design

A prospective randomized placebo‐controlled cohort study was conducted between 2019 and 2022, using foals <6 months of age presented for treatment of diarrhea and systemic inflammation at 3 veterinary hospitals (Veterinary Clinical Centre Charles Sturt University, Scone Equine Hospital, South Eastern Equine Hospital). Foals were excluded if there was evidence of abdominal pain, ileus, or gastric reflux. Foals were randomly allocated by ballot into a control or treatment (FMT) group. Foals in the FMT group were administered 200 to 400 mL (4‐6 mL/kg) of freshly prepared FMT by nasogastric intubation once daily for 3 days. Foals in the control group received 200 to 400 mL of isotonic electrolyte solution once daily for 3 days. All foals were administered omeprazole (nonenteric coated 4 mg/kg or enteric‐coated 2 mg/kg) per os at least 60 minutes before FMT or electrolyte administration.

Feces or fecal swabs were collected 3 to 5 minutes before treatment on Day 0 (D0) and on D1, D2, D3, and D7. Once collected, samples were stored at −20°C until DNA extraction. Samples were analyzed for infectious causes of diarrhea: PCR was used to test for *Salmonella* spp., *C. difficile* toxins A and B genes, *C. perfringens* toxin A, CPE, cpb2 and netF genes, *Cryptosporidium* spp, equine coronavirus, and equine rotavirus A (Vetnostics, ASAP Laboratory), and enrichment and selective culture methods were used to test for *Salmonella* spp. (Veterinary Diagnostics Laboratory Charles Sturt University, ASAP Laboratory, Scone Equine Hospital Laboratory). Fecal smears were also used to confirm *Cryptosporidium* spp. (Scone Equine Hospital Laboratory).

Animal age, sex, breed, current comorbidities, and duration of diarrhea before enrolment were recorded. Clinical data including demeanor, fecal consistency, heart rate, respiratory rate, rectal temperature, mucous membrane color, and capillary refill time were recorded at admission and the results of daily clinical examinations were recorded. Results of hematological and serum biochemical examinations (including serum amyloid A and fibrinogen) were recorded on D0, D1, D2, D3, and D7. Outcome data consisted of time to resolution of diarrhea and survival to discharge from hospital.

Throughout the study, foals received treatment for diarrhea and systemic inflammation at the discretion of the attending veterinarians and according to veterinary hospital protocols. This research was approved by the Animal Care and Ethics Committee of Charles Sturt University (Protocol number A19299). Owner consent was obtained before enrolment in the study.

### 2.2 Donor horses

One donor horse was recruited at each veterinary hospital. Donor horses were determined to be healthy based on physical examination and underwent screening yearly for infectious agents including *C. difficile*, *C. perfringens*, equine coronavirus, *L. intracellularis*, and *Salmonella* spp. by PCR analysis (Vetnostics, Australia). Cultured donor feces were assessed for extended spectrum B lactamase (ESBL) producing *Escherichia coli* isolates using the Cefpodoxime Combination Kit (Thermofisher Scientific, Australia). Organisms were interpreted as containing an ESBL if zone size was increased ≥5 mm between the combination disk compared with cephalosporin alone (Veterinary Diagnostic Laboratory, Charles Sturt University).

### 2.3 FMT preparation

Fresh manure was collected from donor horses by rectal evacuation, and FMT solution was prepared 15 minutes before treatment each day. Approximately 300 g of manure was combined with 1 L of warm chlorinated tap water (approximately 95°F) and macerated using an immersion blender for 30 to 60 seconds to facilitate release of bacteria from fecal particulate matter. The preparation was then strained through a wire strainer or 4 cm × 4 cm gauze swabs, and collected into a clean container.

### 2.4 DNA extraction and next‐generation sequencing

Each fecal sample was thawed for 10 minutes in a warm water bath (approximately 30°C/86°F), and DNA was extracted using Quick‐DNA Fecal/Soil Microbe Miniprep Kit (Zymo Research, CA). Cotton tips of rectal swabs were placed into a bead‐beating tube with lysis buffer, and bead beating was performed at 2800 rpm for 5 minutes using the Digital Disruptor Genie (Scientific Industries, New York, USA). Poor DNA yield and quality were identified at the initiation of DNA extraction of particular batches of samples, and 100 μL of sterile poly‐buffered saline was added to the cotton tip of some swabs before bead beating because of poor DNA yield and quality. The remainder of the extraction was performed as per the manufacturer's instructions. Concentration and purity were assessed by NanoDrop 2000 Spectrophotometer (Thermo Scientific, Australia). DNA was stored at −20°C for 8 weeks before submission for sequencing.

For each sample, the extracted DNA underwent 16S rRNA sequencing at Novogene sequencing facility (Singapore). Samples were filtered, buffered, washed, and eluted as per product protocol. Prior to sequencing, all samples underwent quality control. PCR amplification was performed on the V3‐V4 regions with primer sequence CCTAYGGGRBGCASCAG‐GGACTACNNGGGTATCTAAT. To select PCR products of appropriate size, 2% agarose gel electrophoresis was used. The products were then pooled, end‐repaired, A‐tailed, and ligated with Illumina adapters. Library sequencing was undertaken on a paired‐end Illumina platform to generate 250 bp paired‐end raw reads.

### 2.5 Analysis of sequence data

Raw data were spliced and filtered to acquire clean data. Sequences from the clean data with abundances <5 were filtered out using DADA2^25^ to obtain the final amplicon sequence variants (ASVs). The Quantitative Insights Into Microbial Ecology (QIIME2) algorithm was used for species annotation of the ASV.^26^, ^27^ The representative sequence of each ASV was annotated using QIIME2 software. Relative abundance of phylum, class, order, family, and genus taxonomic levels were determined for the FMT, control, and donor groups. For each group, alpha diversity measures of richness (Chao1, Observed_otus), evenness (Shannon's index), and diversity (Simpson's index of diversity) were generated in QIIME2.^28^ Beta diversity was assessed with weighted and unweighted UniFrac distance metric,^29^, ^30^, ^31^ and visualized using principal coordinate analysis plots generated in QIIME2.

### 2.6 Statistical analysis

Continuous clinical and clinicopathological data were assessed for normality by the Shapiro‐Wilk test.^32^ Nonnormally distributed data were log transformed. Comparisons between treatment and control groups were performed using independent *t*‐tests and Mann‐Whitney tests for normally distributed and nonnormally distributed variables, respectively. Proportions were compared between groups using Fisher's exact or Chi‐square test. Repeated measures for continuous variables from Day 0 to 3 inclusive were analyzed by repeated measures analysis of variance with post hoc testing by Tukey test or, for nonparametric data, Friedman's test and post hoc analysis with Dunn's tests were used. Analyses were performed using commercial software (Prism version 9.5.0, GraphPad Software Inc, San Diego, CA, USA). For all analyses, significance was set at *P* ≤ .05.

To compare the relative abundances of ASV, independent *t*‐tests were performed using R Software (version 3.5.3). Multiple comparison testing of *t*‐test results was performed using Benjamin and Hochberg's false discovery rate to provide adjusted *P*‐values.^33^ Taxa enriched in the fecal samples of each foal group were determined using linear discriminant analysis effect size (LEfSe),^34^ based on *P* < .05, and a linear discriminate analysis score > 4. For LEfSe analysis, LEfSe software (version 1.0) was used. Alpha diversity was assessed using the ASV annotated by QIIME2 to result in Observed features, Chao1, Shannon and Simpson indices, and Kruskal‐Wallis^35^ and Tukey tests were used to compare between groups and time points. Beta diversity was calculated based on weighted and unweighted Unifrac distances.^29^, ^30^ Principal coordinate analysis was performed to visualize differences of samples in complex multidimensional data. To assess differences in community structure between groups, analysis of similarity test (ANOSIM) was performed by the ANOSIM function in QIIME2 software.^36^

## 3 RESULTS

### 3.1 Clinical data

Twenty‐five foals were enrolled in the study (Veterinary Clinical Centre (13), South Eastern Equine Hospital (3) and Scone Equine Hospital (9)), including Thoroughbred (13), Standardbred (4), pony (2), Warmblood (2), Quarter horse (2), Australian stockhorse (1), and Arabian (1) foals. There were 16 colts and 9 fillies. The median age of the foals was 45 days (range, 3‐122). The duration of diarrhea before presentation ranged from 0 to 22 days. Treatment that foals had received before enrolment included intravenously and enterally administered fluids, anti‐inflammatory medication, intestinal adsorbents, gastrointestinal protectants, nutritional support and antimicrobial drugs. Twelve and eight foals were administered antimicrobial drugs and nonsteroidal anti‐inflammatory drugs, respectively, before enrolment into the study. Treatments that foals in the FMT and Control groups received during the study period included antimicrobial drugs (n = 24), butorphanol (n = 3), di‐tri‐octahedral smectite (Bio‐Sponge) (n = 16), bio‐absorbent clay (Bioclay) (n = 1), sucralfate (n = 11), polyionic crystalloid fluids administered intravenously (n = 21), plasma (n = 9), polyionic crystalloid fluids administered enterally (n = 5), electrolyte paste administered enterically (n = 9), lactase (n = 10), hypertonic saline (n = 1), meloxicam (n = 4), flunixin meglumine (n = 11), phenylbutazone (n = 1), yoghurt (n = 1), misoprostol (n = 2), parental nutrition (n = 4), natural colloidal volcanic minerals (n = 2), lignocaine continuous rate infusion (n = 1) and paracetamol (n = 1). Antimicrobial drugs that were used include gentamicin (n = 13), metronidazole (n = 7), cefazolin (n = 7), benzyl penicillin (n = 5), ceftiofur (n = 5), procaine penicillin (n = 4), oxytetracycline (n = 3), sulfadiazine‐trimethoprim combination (n = 2), ceftriaxone (n = 2), clarithromycin (n = 2), rifampin (n = 2), doxycycline (n = 2), and azithromycin (n = 1). Sixteen foals in the FMT group and 8 foals in the Control group received antimicrobial drugs during the study period. Duration of antimicrobial drug treatment ranged from 2 to 7 days and the number of antimicrobial drugs that a single animal received during hospitalization ranged from 0 to 4. There was no difference between the FMT and control groups for age (*P* = .5), sex (*P* = .9), duration of diarrhea (*P* = .1), antimicrobial administration (*P* = .9), or veterinary hospital (*P = *.8).

Sixteen foals were allocated to the FMT group, and 9 foals were allocated to the Control group. Three foals initially enrolled in the Control group were subsequently enrolled in the FMT group because of the persistence of diarrhea. Persistence of diarrhea was defined as the presence of diarrhea for 4 days or longer after initial enrolment into the Control group.

**FIGURE 1:**
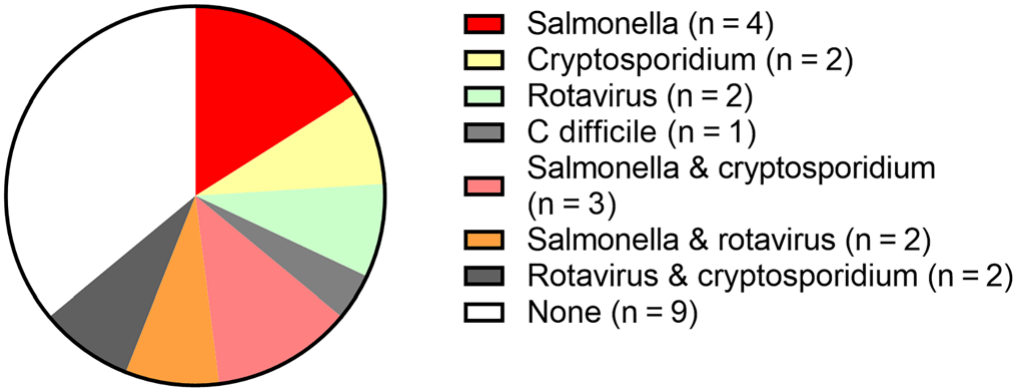
Pie chart displaying the identified enteropathogens from foals with diarrhea.

Comorbidities were present in 11 foals (44%) at the time of presentation: pneumonia (n = 4), prior abdominal surgery for intestinal disease (n = 2), corneal ulceration (n = 1), neonatal isoerythrolysis (n = 1), dorsal displacement of the soft palate (n = 1), osteomyelitis (n = 1), and uroperitoneum (n = 1). Enteric pathogens were identified in 16/25 (77%) of foals: *Salmonella* spp. (n = 9); *rotavirus* A (n = 9); C*ryptosporidium* spp. (n = 7) and *C. difficile* (n = 1) (Figure 1). Seven foals had multiple pathogens identified. No enteropathogens were detected in 9 foals.

On D0, there were no differences in clinical, hematological, or blood biochemical variables between groups, with the exception of mildly increased respiratory rate in the Control group. There was a reduction in heart rate over time in the FMT group (*P* = .002), with differences between D0 and D2 (*P* = .005) and D0 and D3 (*P* < .001). By contrast, no reduction in heart rate was evident in the Control group (*P* = .07). Over time, there was no difference in respiratory rate in the FMT group (*P* = .46), while there was a reduction in respiratory rate in the control foals (*P* = .03), but differences between time points were not significant. There was no difference in rectal temperature between groups. Clinical and clinicopathological data are presented in Table S1.

**FIGURE 2:**
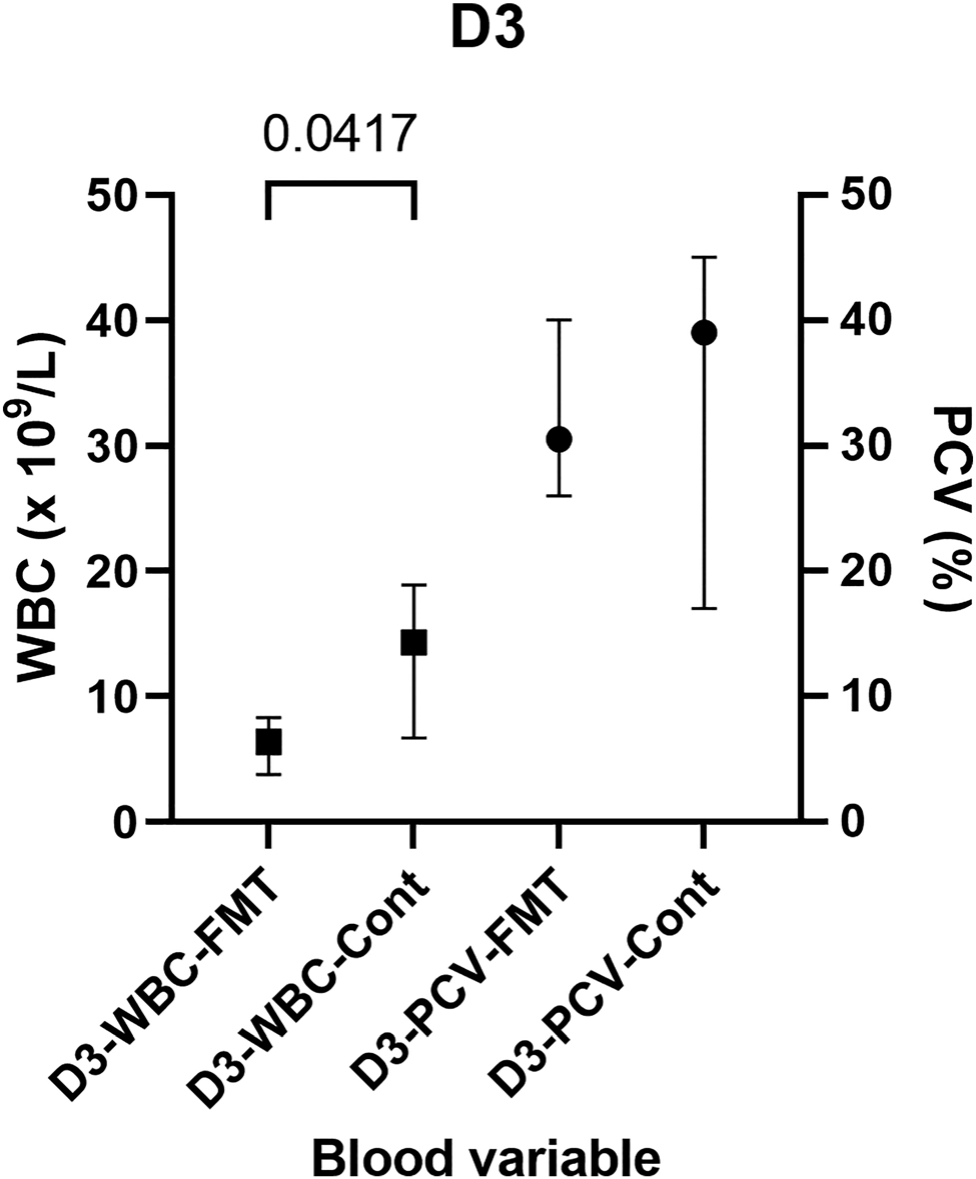
WBC and PCV of the FMT group and Control group on D3. FMT, fecal microbiota transplantation; WBC, white blood cell.

On D3, the white blood cell (WBC) count of the FMT group was lower than that of the Control group (*P* = .04) (Figure 2). The PCV of the FMT group was lower than that of the Control group on D3; however, this finding was not significantly different (*P* = .09) (Figure 2). In the FMT group, the PCV reduced over time and was lower on D3 compared to D0 (*P* = .04). A corresponding decrease in PCV was not observed in Control group foals (*P* = .4).

The concentration of chloride in serum increased over time in the FMT group (*P* = .05), with a mean difference between D0 and D1 of 4.5 mmol/L (95% CI, 0.5‐8.5; *P* = .03). A change in serum chloride concentration was not observed in the Control group.

**FIGURE 3:**
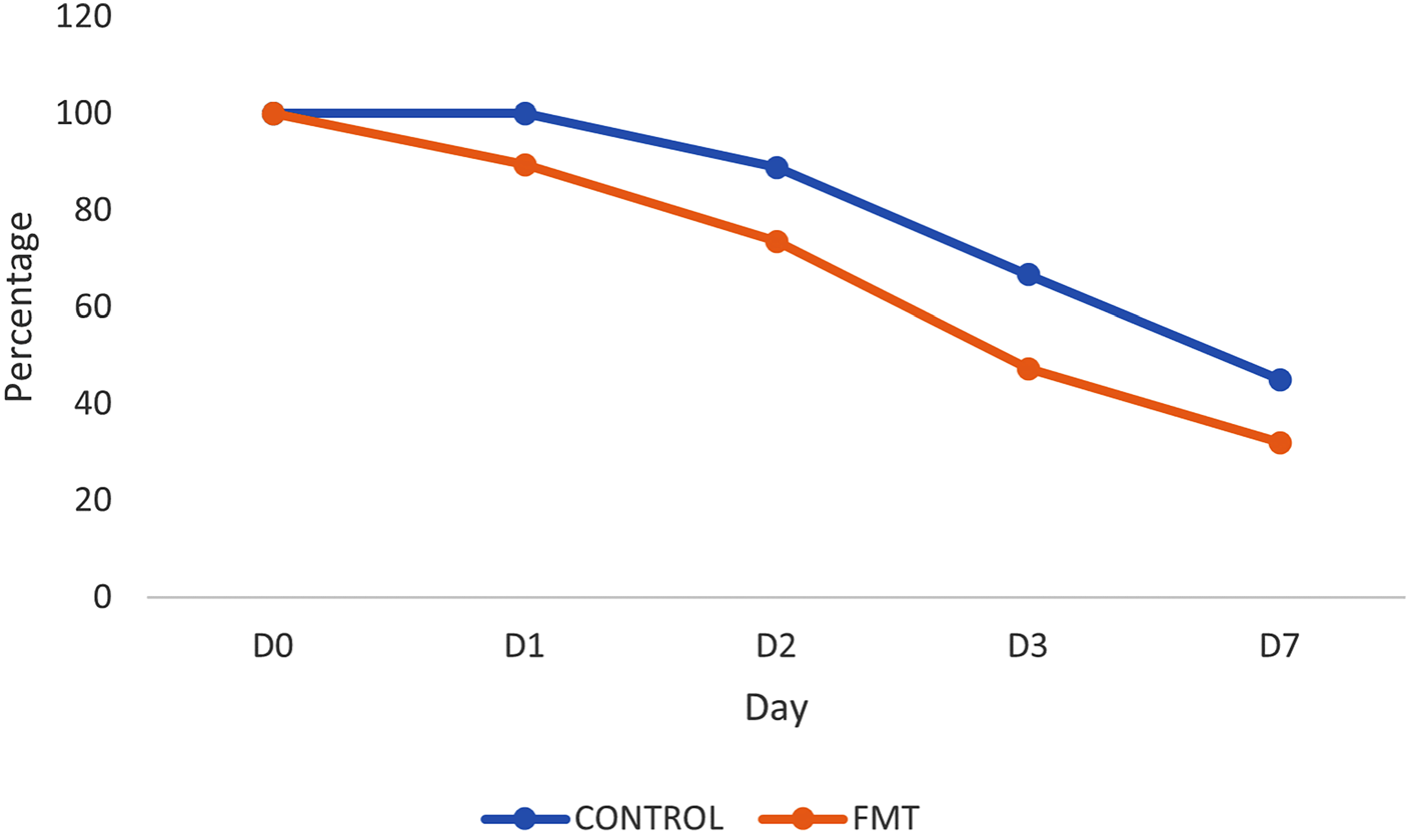
Line graph displaying the prevalence of diarrhea within the FMT group and the control group during the study period. FMT, fecal microbiota transplantation.

Resolution of diarrhea within 7 days of initiation of treatment was more often observed in the FMT group (13/19, 68%) than in the Control group (4/9, 55%) (*P* = .4); however, differences were not significant (Figure 3). There was no difference in survival to discharge between the FMT (15/19, 79%) and Control groups (9/9, 100%) (*P* = .3).

### 3.2 Sequence analysis

Feces from 2 of the 3 donor horses were obtained for microbiota analysis. The 106 samples from control foals (n = 34), FMT foals (n = 70), and donors (n = 2) yielded 3 992 044, 9 099 602, and 262 199 raw reads, respectively, with a total yield of 13 353 765. Quality filtering and primer sequence removal resulted in 11 253 352 qualified reads that were used for analysis.

### 3.3 Relative abundance

**FIGURE 4:**
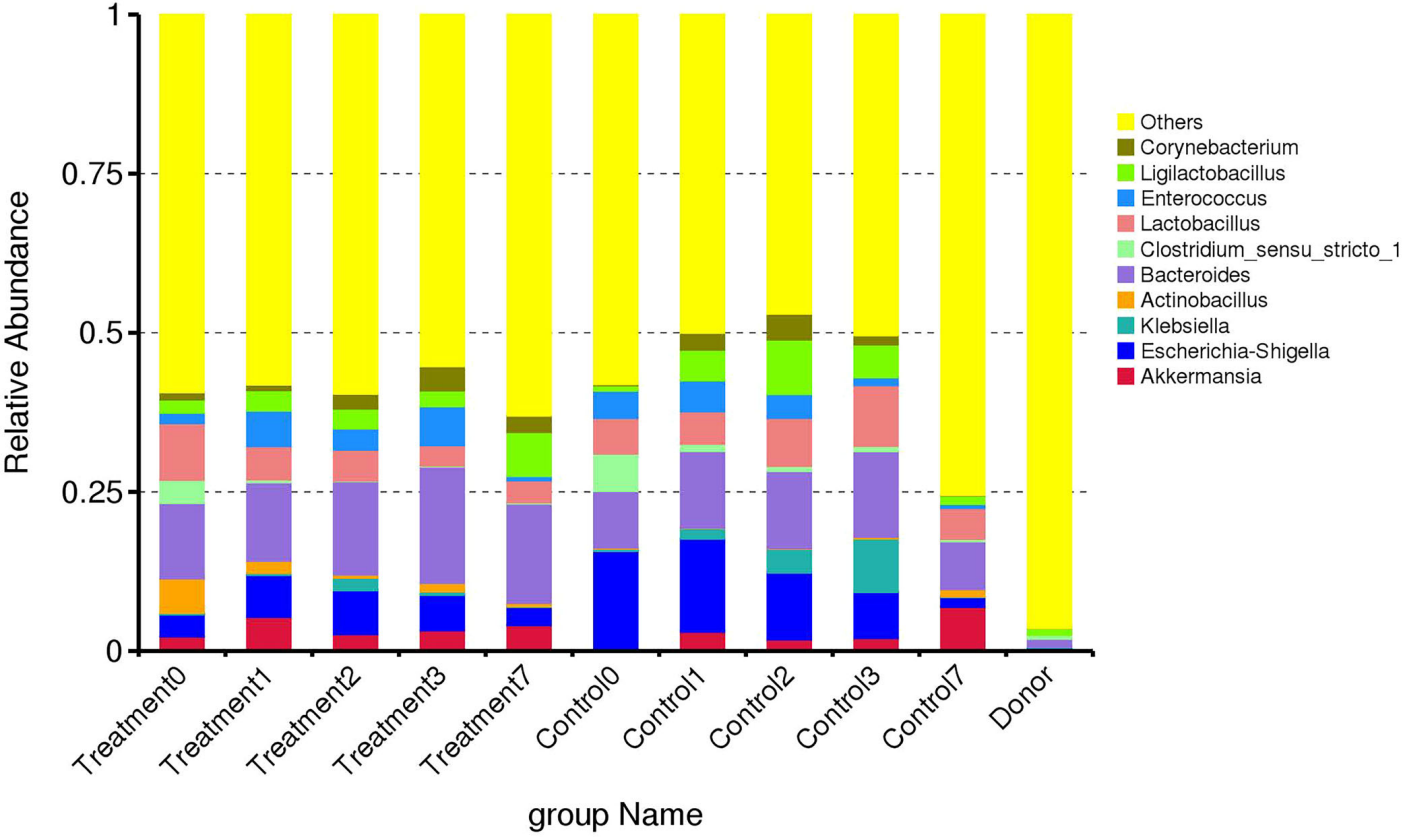
The fecal microbiota relative abundance at genus level. The bar chart shows the relative abundance of the donor horses, and each foal group at each sampling time point.

The ASVs were clustered and allocated to levels of taxonomic classification including 31 phyla, 126 orders, 59 classes, 220 families, and 443 genera. The 5 most abundant phyla contributed 95.9% of the identifiable ASVs in the foals and included Firmicutes (54.4%), Bacteroidota (22%), Proteobacteria (12.6%), Fusobacteroita (3.6%), and Actinobacteroita (3%). The 5 most abundant phyla in the donor horses contributed 95.8% of the identified ASVs and included Firmicutes (69.4%), Bacteroidota (18.8%), Spirochaetota (4.5%), Fibrobacter (2%), and Verrucomicrobiota (1.1%). On D0, the 10 most abundant genus in the foals included *Bacteroides* (12.7%), *Escherichia‐Shigella* (7.4%), *Lactobacillus* (5.8%), *Ligilactobacillus* (3.8%), *Enterococcus* (3.2%), *Akkermansia* (3.2%), *Corynebacterium* (1.9%), *Klebsiella* (1.7%), *Clostridium_sensu_stricto_1* (1.3%), and *Actinobacillus* (1.1%) (Figure 4). In the donor horses at D0, the 10 most abundant genus were *Christensenellaceae_R‐7_group* (10.3%), *NK4A214_group* (6.1%), *Treponema* (4.5%), *UCG‐005* (3.5%), *Lachnospiraceae_XPB1014_group* (3.4%), *Rikenellaceae_RC9_gut_group* (3%), *Fibrobacter* (2%), *UCG‐002* (2%), [*Eubacterium]_hallii_group* (1.8%), and *Blautia* (1.6%).

There were no differences in the relative abundance at the phylum or genus taxonomic level between FMT and control foals at each sampling time point, or within groups over time. There were differences in class Negativicutes (*P* = .05) and families *Enterobacteriaceae* (*P* = .05), *Veillonellaceae* (*P* = .05), *Burkholderiaceae* (*P* = .05) and *Streptococcaceae* (*P* = .05) between the FMT and Control group at D0. In the FMT group over time, there was an increase in order Rhizobiales (*P* = .05) at D0 compared to D3. Differences in relative abundances between and within groups are presented in Table S2.

**FIGURE 5:**
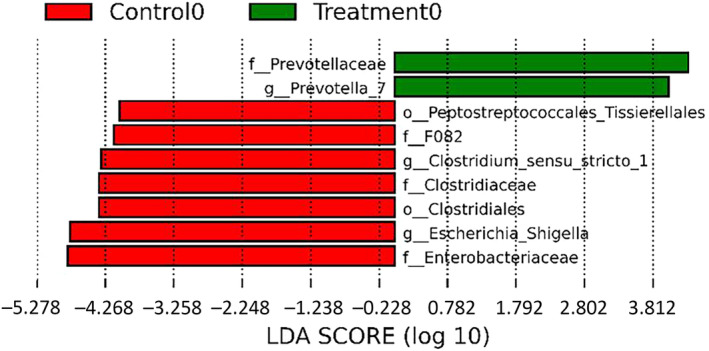
The enriched fecal microbiota displayed by LEfSe histogram, which shows a number of differences in taxa at D0. LEfSe, linear discriminant analysis effect size.

**FIGURE 6:**
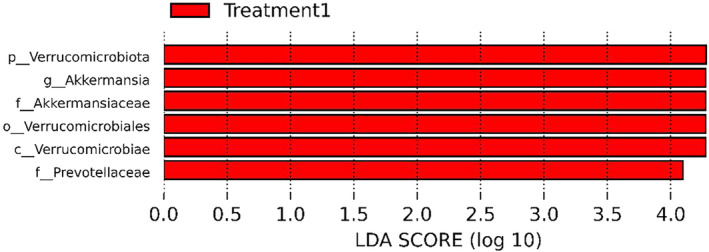
The enriched fecal microbiota displayed by a LEfSe histogram, which shows enriched features in the FMT group at Day 1 compared with the Control group on Day 1. FMT, fecal microbiota transplantation; LEfSe, linear discriminant analysis effect size.

LEfSe analysis revealed differences in the enrichment of orders, families, and genera at D0 between the Control and FMT groups before treatment (Figure 5). On D1, a number of taxa were enriched in the FMT group compared to the Control group, including Phylum Verrucomicrobiota, genus *Akkermansia*, and family *Prevotellaceae* (Figure 6).

### 3.4 Alpha diversity measures

**FIGURE 7:**
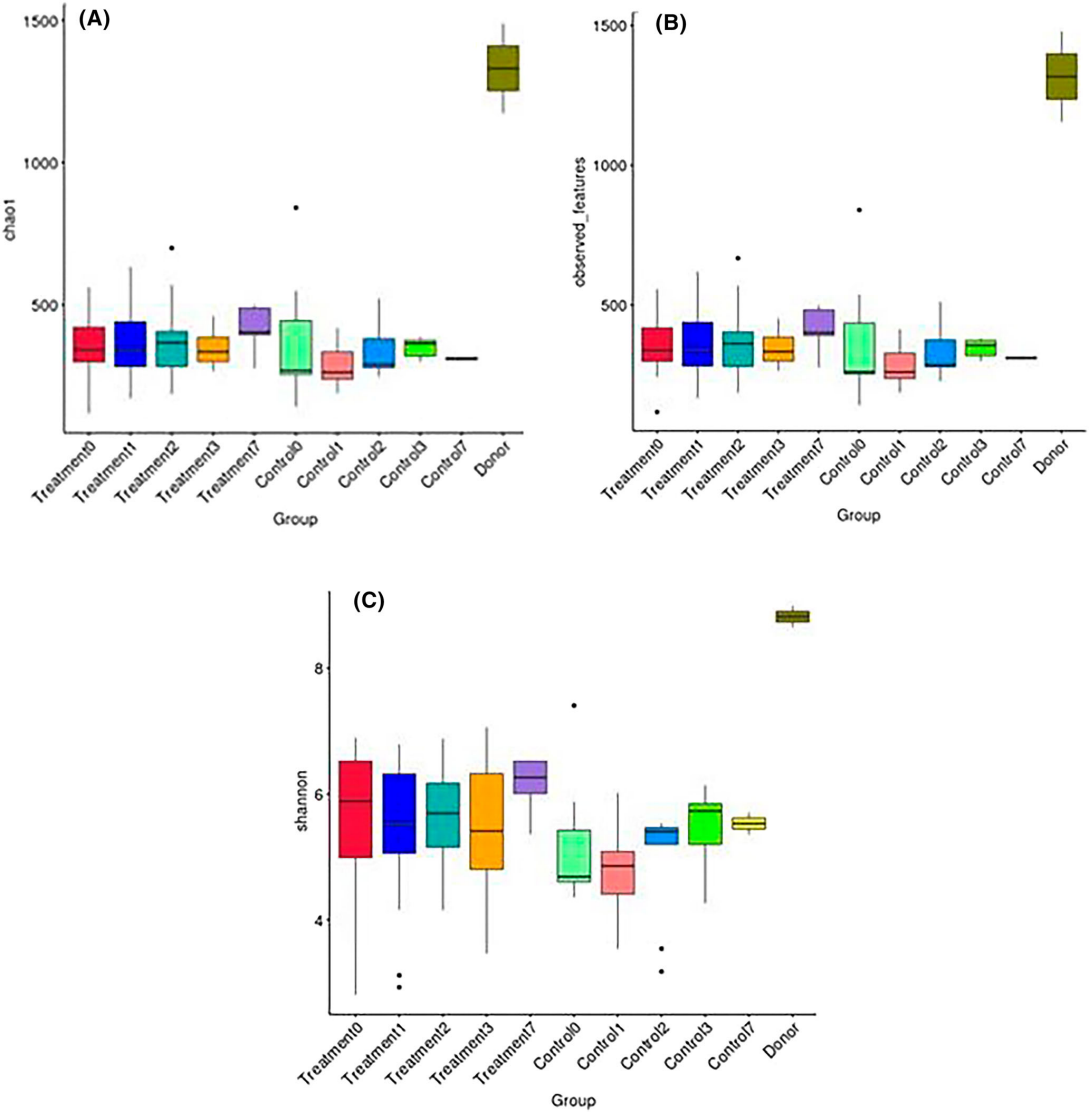
Measures of alpha diversity including Chao1 (A) observed features (B) and Shannon (C) box plot displaying differences in alpha diversity between groups. The donor horses had significantly increased richness and diversity compared with all foals.

There were no significant differences in alpha diversity between the FMT and Control group at D0, D1, D2, D3, or D7. There was increased richness in donor horses compared to foals (Figure 7A,B).

### 3.5 Beta diversity measures

**FIGURE 8:**
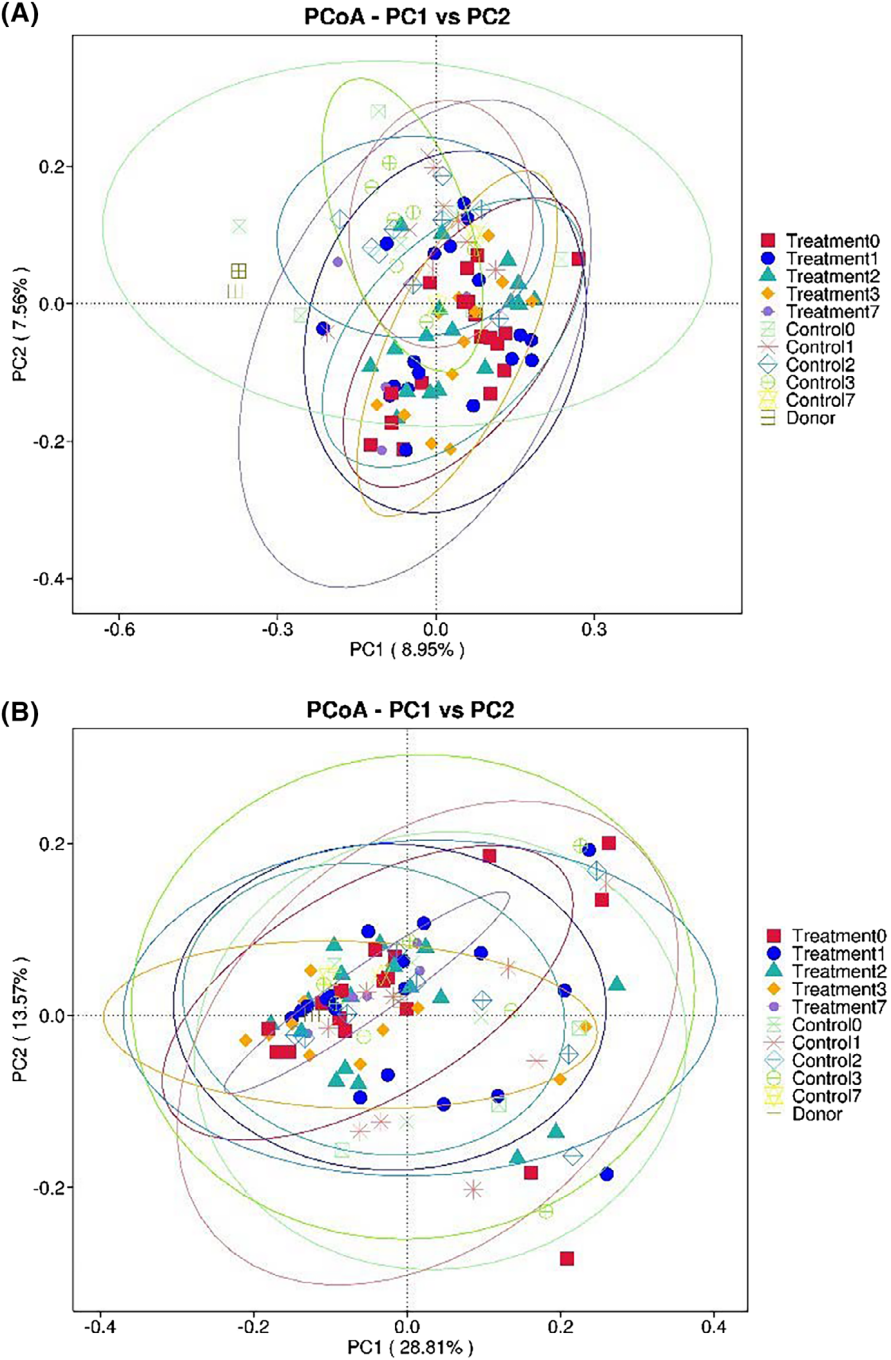
Weighted (A) and unweighted (B) principal coordinate analysis of the fecal microbiota of foals with diarrhea and adult donor horses. Each shape represents each group at each time point during the study period.

Principal coordinate analysis analyses of unweighted and weighted UniFrac distances revealed considerable overlap between FMT and Control groups over all time points (Figure 8) indicating similarities in bacterial community membership and structure (ANOSIM, *P* = .1‐1).

## 4 DISCUSSION

In this study, the effect of FMT in foals with diarrhea was assessed. Administration of FMT was associated with the improvement of some clinical and clinicopathological variables. There were no differences in the relative abundance of phylum or genus between the FMT and Control group; however, there was enrichment of phylum Verrucomicrobiota and genus *Akkermansia* in the FMT group at D1. There were no differences in alpha diversity within or between groups over time. Although the resolution of diarrhea and survival to discharge were not significantly different between groups, a beneficial effect of FMT treatment cannot be discounted. The concurrent administration of other medications, most importantly antimicrobials, could have precipitated persistent dysbiosis and had an effect on outcome variables.

Foals receiving FMT evidenced reduced heart rate, WBC and PCV, and increased serum chloride concentrations, relative to pretreatment values or corresponding results from control foals. Reductions in PCV and heart rate could be associated with improved hydration and improved hemodynamic status in treated animals. Increased chloride concentrations at D1 compared with D0 could reflect decreased mucosal secretion and improved absorptive capacity associated with resolving intestinal damage. This could have resulted from improvement of the integrity and function of the intestinal mucosa by reestablishment of the intestinal microbiota. The decreased WBC count in the FMT group, compared with control foals at D3, suggests reduced inflammation in treated foals. The gastrointestinal microbiota has an important role in metabolic and immune function in the host,^37^, ^38^ and it is possible that the administration of FMT assisted in the restoration of local immune responses in those foals.

In this study, there was no difference in resolution of diarrhea or survival between the 2 groups. There was no evidence that FMT exacerbated diarrhea or had a negative effect on outcome. Of the 3 foals initially enrolled into the Control group and then reenrolled into the FMT group, 2 had resolution of diarrhea after FMT. This could be because of continued supportive care and time; however, this finding could reflect a beneficial effect of FMT on the intestinal microbiota and intestinal function. Clinical indicators of response to FMT in adult horses include improved fecal consistency^6^, ^7^, ^22^ and resolution of diarrhea^39^; however, studies of adult horses with diarrhea have demonstrated conflicting responses to FMT. Normalization of fecal microbiota has been associated with FMT in 3 of 5 geriatric horses with diarrhea.^20^ In a study of 4 adult horses that developed diarrhea postlaparotomy, FMT was associated with the resolution of pyrexia and diarrhea within 24 hours of the first treatment.^39^ However, that study did not report on the fecal microbiota of recipients^39^ and neither study included a control group. Other studies assessing the response of FMT treatment in adult horses report no improvement in diarrhea,^22^, ^23^ resolution of free fecal water syndrome,^24^ or differences in clinical and clinicopathological findings compared to control horses.^7^

In the current study, a major effect of FMT on the relative abundance of taxa in the fecal microbiota was not observed. The microbiota of horses is affected by environmental factors including season, feed types, weather conditions,^40^ and geographic location.^7^ Importantly, the fecal microbiota of foals changes dramatically within the first 6 months of life.^11^ The microbiota of foals is different from adult horses and gradually becomes similar to that of mares at the time of weaning.^11^, ^41^ Phyla including Firmicutes, Bacteroidetes, and Verrucomicrobia are more enriched at 2 months of age compared with 1 day of age, reflective of a higher forage‐based diet in older foals.^40^ In the current study, it was not possible to control for age, and this might have contributed to differences in taxa observed at D0 in FMT and control foals, although there was no significant difference in foal age between groups.

In the current study, FMT was associated with the enrichment of phylum Verrucomicrobiota, genus *Akkermansia*, and family *Prevotellaceae*. Genus *Akkermansia* is associated with the maintenance of the integrity of the mucin layer of the intestinal tract and reportedly decreases bowel inflammation in people.^42^ In addition, members of the *Prevotellaceae* family have anti‐inflammatory and modulating effects on the intestines, and have a role in maintaining intestinal health.^43^ The enrichment of this family supports the possible establishment of a more normalized gastrointestinal microbiota and assistance in immune modulation. The gastrointestinal microbiota has an important role in metabolic and immune function in the host,^37^, ^38^ and it is possible that the administration of FMT assisted in the restoration of local immune responses in those foals. The significant differences in enrichment and relative abundance detected at D0 likely reflect the individual variation of the microbiota and introduce difficulty in the interpretation of the changes observed over time.

In our study, alpha diversity of foals was less than donors. This finding is consistent with the findings of previous studies where the alpha diversity of the adult horse fecal microbiota was greater than foals, and increasing alpha diversity in foals was correlated with age.^11^, ^44^ Foals with diarrhea have a lower richness compared to healthy age‐matched peers, represented by lower Chao index^10^ and decreased alpha diversity has been associated with gastrointestinal disease in adult horses.^45^ Studies assessing FMT in adult horses with diarrhea have reported increased alpha diversity^6^, ^7^ and normalization of microbiota scores.^7^ In contrast, other studies reported no change in alpha diversity after administration of FMT to horses with diarrhea of free fecal water syndrome.^22^, ^24^ In the current study, FMT administration was not associated with changes in alpha diversity.

In our study, ANOSIM analysis revealed no differences in beta diversity. The lack of difference could be because of the small group size, the absence of change in the distal intestinal tract, or limitations in using fecal microbiota to characterize the microbial population within the gastrointestinal tract. In one study, administration of FMT to adult horses with diarrhea was associated with changes in beta diversity,^7^ while in other studies, there were no changes in beta diversity associated with treatment with FMT.^22^, ^24^

Foals in this study were administered isotonic polyionic fluids and plasma intravenously, antimicrobial drugs, anti‐inflammatory drugs, intestinal adsorbents, and nutritional care before study enrolment. The administration of FMT could have facilitated improved hemodynamic status by promoting the reestablishment of the intestinal microbiota and restoration of the integrity and function of the intestinal mucosa. However, the likely negative effect of concurrent antimicrobial drug administration on the efficacy of FMT cannot be ignored. Administration of antimicrobial drugs causes changes to the fecal microbiota^13^, ^15^, ^46^ and can lead to dysbiosis and diarrhea.^23^, ^47^ Antimicrobial drugs commonly administered to horses, including metronidazole,^9^, ^14^, ^23^ penicillin,^9^, ^46^ gentamicin,^9^ ceftiofur,^9^, ^15^ trimethoprim‐sulfadiazine combination,^15^ doxycycline,^48^ and enrofloxacin^13^ affect the fecal microbiota in adult horses. Significant changes in taxa including phyla Spirochaetes, Lentisphaerae, Fibrobacteres, and Verrucomicrobia and family *Lachnospiraceae*, and significant reductions in alpha diversity^15^, ^46^, ^49^ and changes in beta diversity^23^, ^46^ are associated with antimicrobial administration.^14^, ^46^, ^48^, ^49^ In the current study, the limited changes to the fecal microbiota and similar clinical results in the FMT and control groups could have been influenced by the concurrent administration of antimicrobial drugs. In foals with diarrhea, it is possible that FMT did not address the underlying antimicrobial‐associated dysbiosis,^25^ or the continued administration of antimicrobials adversely impacted on FMT efficacy.

The current study was a multicenter, prospective randomized clinical trial. The number of animals enrolled is similar to or greater than reports in adult horses^6^, ^7^, ^22^, ^23^; albeit modest. As expected in prospective clinical studies, it was not possible to standardize concurrent veterinary treatments required for animal care, and these treatments could have influenced outcomes in these foals. Some clinical and microbiota data were not available, influenced by individual animal needs and owner decisions, resolution of diarrhea, and discharge from hospital. Inclusion of animals in 3 veterinary hospitals in differing locations could have confounded the results, as it has been demonstrated that geography also influences the gastrointestinal microbiota.^9^ Donor horses were used over a 3‐year period, and changes in the microbiota of these animals might have occurred during this time.

In conclusion, this study demonstrated that FMT in foals is safe and did not exacerbate the occurrence of diarrhea or clinical disease in treated foals. Although survival and resolution of diarrhea were similar in both groups, there was evidence to suggest that foals treated with FMT had improvement in some clinical and clinicopathological findings, and a beneficial effect of FMT on the gastrointestinal microbiota cannot be discounted. Further studies assessing the outcomes associated with FMT in foals and preparation protocol would further refine treatment recommendations in the future.

## CONFLICT OF INTEREST DECLARATION

Kristopher J. Hughes serves as Associate Editor for the Journal of Veterinary Internal Medicine. He was not involved in review of this manuscript. No other authors declare a conflict of interest.

## OFF‐LABEL ANTIMICROBIAL DECLARATION

Off‐label antimicrobials cefazolin, gentamicin and ceftiofur were administered to foals in this study.

## INSTITUTIONAL ANIMAL CARE AND USE COMMITTEE (IACUC) OR OTHER APPROVAL DECLARATION

Approved by the Charles Sturt University Animal Care and Ethics Committee (Protocol number A19299).

## HUMAN ETHICS APPROVAL DECLARATION

Authors declare human ethics approval was not needed for this study.

## Supporting information


**Data S1.** Supporting Information.
