# Supplementary material for: Effects of fecal microbiota transplantation on clinical outcomes and fecal microbiota of foals with diarrhea
Source: J Vet Intern Med. 2024 Sep 12;38(5):2718–28. doi: 10.1111/jvim.17185 (PMC11423448; doi:10.1111/jvim.17185)
Supplement: Supplementary file 1 — Data S1. Supporting Information. [file JVIM-38-2718-s001.docx]

**Supplementary Table 1**

Table 1: Comparison of clinical and clinicopathological findings between Control and faecal microbiota transplantation (FMT) groups throughout the study period. Results are presented as median (range) or mean ± standard deviation.

| Variable | D0 | | D1 | | D2 | | D3 | | D7 | |
| --- | --- | --- | --- | --- | --- | --- | --- | --- | --- | --- |
|  | Control  (*n*=9) | FMT  (*n*=19) | Control  (*n*=9) | FMT  *(n*=19) | Control  (*n*=9) | FMT  (*n*=19) | Control  (*n*=9) | FMT  (*n*=19) | Control  (*n*=6) | FMT  (*n*=9) |
| Heart Rate (bpm) | 88  (76-124) | 80  (60-150) | 68  (52-100) | 74  (56-120) | 88  (50-156) | 70  (52-110) | 68  (44-104) | 64  (54-102) | 60  (48-100) | 60  (60-80) |
| Respiratory Rate (brpm) | 28^*^  (16-52) | 20  (12-44) | 20  (12-40) | 23  (12-40) | 24  (12-32) | 20  (12-40) | 20  (16-28) | 20.5  (16-44) | 24  (16-40) | 28  (12-40) |
| Temperature (^o^F) | 100.9  (100.5-103.6) | 101.5 (99.9-104.5) | 100.2  (99.5-101.1) | 100.9  (99-102.2) | 100.8  (99.1-101.8) | 100.8  (99-102.6) | 100.8  (99.1-101.5) | 100.2  (98.8-103.5) | 101.1  (100.4-103.5) | 100.6  (99.5-101.8) |
| White blood cells (g/L) | 7.2  (2.7-13.5) | 8.2  (1.9-21.1) | 8.7 (8.7-8.7) | 11.2 (10.6-11.8) | 7 (7-7) | 13.1 (2.7-15.6) | 14.3^*^  (6.7-18.9) | 6.4  (5.0-8.3) | NA | NA |
| Neutrophil (g/L) | 3.6  (0.2-11.2) | 5.3  (0.7-14.5) | NA | NA | NA | NA | 7.6  (4.9-16.6) | 4.4  (3.7-6.1) | NA | NA |
| PCV (%) | 36  (20-46) | 35  (29-55) | 36  (17-37) | 34  (26-48) | 34.5  (24-46) | 32  (28-41) | 39  (17-41) | 30.5  (29-40) | 38.5  (36-41) | 31  (28-34) |
| TPP (g/dL) | 5.71 ± 0.76 | 5.84 ± 1.34 | 5.12 ± 0.58 | 5.3 ± 1.34 | 5.35 ± 0.7 | 52.0 ±15.2 | 5.23 ± 0.55 | 4.67 ± 1.55 | 5.97 ± 0.65 | 5.1 ± 0.65 |
| SAA (mg/L) | 1216  (0.73-2719) | 503  (3-2096) | NA | NA | NA | NA | NA | NA | NA | NA |
| Sodium (mEq/L) | 130  (123-137) | 131  (112-137) | 130  (129-135) | 131.5  (119-146) | 129.5  (115-137) | 134  (127-145) | 133  (124-140) | 132.5  (125-135) | NA | NA |
| Chloride (mEq/L) | 96.1 ±6.3 | 95.6 ± 6.5 | 96.8 ± 2.3 | 100 ±6.4 | 95.5 ± 4.4 | 102.6 ± 5.8 | 99 ± 4.8 | 97.8 ± 5.8 | NA | NA |
| Potassium (mEq/L) | 3.3 ± 0.8 | 3.4± 0.9 | 2.9 ± 0.6 | 3.4 ± 0.8 | 3.5 ± 1.1 | 3.3 ± 0.8 | 4.3 ±0.7 | 3.6 ± 0.6 | NA | NA |
| Lactate (mmol/L) | 1.6  (1.1-2.2) | 1.5  (0.6 – 10.5) | 0.9  (0.7-1) | 0.9  (0.5- 1.9) | 1  (0.7-2.5) | 1.1  (0.7 -1.1) | 0.9  (0.9- 0.9) | 1.1  (0.4 – 2.9) | NA | NA |
| Glucose (mg/dL) | 118.9  (90– 189.2) | 122.5  (104.5- 182) | NA | NA | NA | NA | 127.9  (118.9-136.9) | 129.7  (108 -140.5) | NA | NA |
| Creatinine (mg/dL) | 1.18  (0.93 – 1.64) | 1.23  (0.7-2.8) | NA | NA | NA | NA | 1.0  (0.99-1.1) | 1.27  (0.76-1.54) | NA | NA |

Footnote

^*^ represents significant differences between groups ( *P*  ≤ .05)

NA represents time points were inadequate data was obtained

**Supplementary Table 2**

Table 2: Significant differences of relative abundance of phyla, class, order, family, genus and species within and between groups determined by T-test. For comparisons between groups, the relative abundance of the FMT is compared to the control group. Within groups, the relative abundance is for D1, D2 and D3 compared to D0.

| *FMT v Control* | *Phyla* | *Class* | *Order* | *Family* | *Genus* |
| --- | --- | --- | --- | --- | --- |
| D0 | - | Negativicutes (↑) (.05) | - | Enterobacteriaceae(↓)(.05)  Veillonellaceae(↑) (.05) Streptococcaceae(↑)(.05)  Burkholderiaceae (↓) (.05) | - |
| D1 | - | - | - | - | - |
| D2 | - |  | - | **-** | - |
| D3 | - | - | - | - | - |
| *FMT group* |  |  |  |  |  |
| D0-D1 | - | - | - | - | - |
| D0-D2 | - | - | - | - | - |
| D0-D3 | - | - | Rhizobiales(↑)(.05) | - | - |
| *Control group* |  |  |  |  |  |
| D0-D1 | - | - | - | - | - |
| D0-D2 | - | - | - | - | - |
| D0-D3 | - | - | - | - | - |
